# Evaluation of T-NGS, Xpert Ultra, and Line Probe Assay on Clinical Samples for the Diagnosis of Drug-Resistant Pulmonary Tuberculosis in Rio de Janeiro

**DOI:** 10.1590/0037-8682-0358-2025

**Published:** 2025-12-15

**Authors:** Marcela Bhering, Eunice Petris Ribeiro, Anna Karla Silveira, Carolyne Lalucha, Elizabete Oliveira Ribeiro, Tathiany Igreja, Luís Cristóvão Pôrto, Danielle Secco, Pedro Eduardo Almeida da Silva, Miguel Viveiros, Martha Oliveira, Afrânio Kritski

**Affiliations:** 1 Universidade Federal do Rio de Janeiro, Faculdade de Medicina, Programa Acadêmico de Tuberculose, Rio de Janeiro, RJ, Brasil.; 2 Fundação Oswaldo Cruz, Escola Nacional de Saúde Pública Sergio Arouca, Rio de Janeiro, RJ, Brasil.; 3 Universidade do Estado do Rio de Janeiro, Laboratório de Histocompatibilidade e Criopreservação, Rio de Janeiro, RJ, Brasil.; 4 Universidade Federal do Rio Grande, Faculdade de Medicina, Núcleo de Pesquisa em Microbiologia Médica, Rio Grande, RS, Brasil.; 5 Universidade Nova de Lisboa, Instituto de Higiene e Medicina Tropical, Lisboa, Portugal.

**Keywords:** Drug-Resistant tuberculosis, Molecular diagnostic techniques, Sensitivity and specificity, diagnosis. *Mycobacterium tuberculosis*

## Abstract

**Background:**

The rapid detection of drug resistance in *Mycobacterium tuberculosis* is essential for managing drug-resistant tuberculosis (DR-TB). This study evaluated the performance of molecular assays compared to phenotypic drug susceptibility testing (pDST) and targeted next-generation sequencing (T-NGS).

**Methods:**

We retrospectively analyzed 40 presumptive pulmonary DR-TB cases in Rio de Janeiro from 2018 to 2022. Xpert MTB/RIF Ultra (Xpert Ultra) and Line Probe Assay (LPA; MTBDRplus = LPA-1, MTBDRsl = LPA-2) were performed directly on clinical respiratory specimens, with pDST serving as the reference standard. T-NGS was used to identify resistance mutations and clarify discordant results.

**Results:**

Most samples (92.5%) were smear-positive. Xpert Ultra and LPA-1 demonstrated high sensitivity for detecting resistance to rifampicin (91.7% and 89.3%, respectively). However, LPA-1 exhibited lower sensitivity for isoniazid (81.5%). The performance of LPA-1 decreased in samples with cycle threshold (Ct) values ≥16, indicating low bacterial load (p = 0.001). T-NGS detected resistance to fluoroquinolones (22.5%) and injectables (15-20%) that was missed by LPA-2 and MGIT. Mixed infections were identified in 17.5% of samples and accounted for 27.8% of discordant results. Isoniazid heteroresistance was detected in 32.5% of samples by LPA-1 and in 7.5% by T-NGS.

**Conclusions:**

Xpert Ultra and LPA-1 are effective for the rapid detection of rifampicin resistance but have limitations for isoniazid and second-line drugs. T-NGS improved the detection of low-level resistance, heteroresistance, and mixed infections, supporting its implementation in reference laboratories for comprehensive DR-TB diagnosis.

## INTRODUCTION

The emergence of the COVID-19 pandemic in 2020 significantly disrupted tuberculosis (TB) diagnostic services, resulting in documented reductions in diagnosis, treatment, and preventive therapy. Concurrently, between 2020 and 2021, the global burden of drug-resistant TB (DR-TB) increased, contributing to persistent mortality due to delays in diagnosis and the initiation of appropriate therapy^1^. In 2024, an estimated 10.8 million people developed TB, leading to 1.25 million deaths, including 161,000 among people living with HIV (PLHIV), while over 400,000 individuals developed DR-TB. Globally, 3.4% of new TB cases and 18% of previously treated cases are classified as multidrug-resistant TB (MDR-TB), which involves resistance to both rifampicin (RIF) and isoniazid (INH), or as rifampicin-monoresistant (RR-TB)^2^.

Due to the extended turnaround time required for phenotypic drug susceptibility testing (pDST) (exceeding 60 days in solid media and 30 days in liquid media for first-line drugs [streptomycin/SM, INH, RIF, and ethambutol/EMB] and second-line drugs [fluoroquinolones/FQ and amikacin/AM]^3^), the World Health Organization (WHO) has recommended the use of molecular diagnostics to expedite the detection of DR-TB. The Xpert MTB/RIF assay was the first widely adopted test, demonstrating high accuracy for both drug-sensitive TB (DS-TB) and RR-TB^4^. In 2013, the WHO endorsed the use of novel drugs (bedaquiline and delamanid)^5^ and recommended the application of line probe assays (LPA) directly on clinical specimens or culture isolates to detect resistance to first-line (MTBDRplus; LPA-1) and second-line drugs (MTBDRsl; LPA-2)^6^ ^-^ ^7^.

Despite these advancements, by 2016, fewer than one in three MDR/RR-TB cases were diagnosed, revealing a significant gap between global guidelines and real-world implementation^8^. Among the diagnosed cases, 6.2% met the criteria for extensively drug-resistant TB (XDR-TB), defined as MDR-TB with additional resistance to any fluoroquinolones and at least one Group A drug (e.g., bedaquiline or linezolid). High-burden countries, such as Peru and China, have reported rising rates of primary MDR/XDR-TB in patients without prior TB treatment, highlighting the ongoing transmission of resistant bacilli and the limitations of diagnostic approaches that rely solely on risk-factor profiling^9^ ^,^ ^10^.

To bridge these gaps, the WHO has recommended broader molecular tools, including Xpert XDR for the direct detection of INH and second-line resistance in clinical samples^11^, and targeted next-generation sequencing (T-NGS) for early MDR/XDR-TB detection and regimen eligibility^12^. A systematic review conducted in 2024, involving 5,223 samples from 53 countries, confirmed the high accuracy of T-NGS compared to pDST, particularly for first-line drugs and fluoroquinolones, although sensitivity was lower for pyrazinamide, amikacin, and capreomycin^13^. Few studies in high-burden TB settings have combined pDST and T-NGS to resolve discrepancies and characterize resistance mutations overlooked by conventional molecular assays^14^.

In Brazil, 80,012 new TB cases were reported in 2023; however, the implementation of diagnostic tools remains limited. Xpert MTB/RIF Ultra (Xpert Ultra) and culture were performed in only 51.2% and 28.2% of new cases, respectively, while pDST among previously treated patients reached only 51.5%^15^. States such as Rio Grande do Sul and Rio de Janeiro have reported increasing rates of primary MDR/XDR-TB^16^ ^,^ ^17^, underscoring persistent diagnostic challenges in Brazil, including the limited implementation of molecular tools and difficulties in detecting low-level resistance, heteroresistance, and mixed infections. These challenges directly motivate the present study.

This study aimed to evaluate the accuracy of Xpert Ultra and LPA for detecting resistance to first- and second-line anti-TB drugs directly on clinical samples, utilizing MGIT pDST as the reference standard. Additionally, we applied T-NGS (Deeplex® Myc-TB) to characterize resistance profiles and clarify molecular and phenotypic discrepancies, with particular attention to low-level resistance, heteroresistance, and mixed infections.

## METHODS

### Study Design and Setting

This retrospective observational study utilized clinical samples collected from presumptive pulmonary DR-TB cases tested between 2018 and 2022. The samples were examined for resistance to both first- and second-line drugs of *Mycobacterium tuberculosis*. Laboratory analyses were conducted at the Histocompatibility and Cryopreservation Laboratory (UERJ) and the Molecular Mycobacteriology Laboratory (UFRJ), both located in Rio de Janeiro, Brazil.

Archived respiratory clinical specimens from presumptive pulmonary DR-TB cases were included consecutively, only after the completion of all assays, which consisted of Xpert MTB/RIF Ultra, LPA, MGIT pDST, and T-NGS [Deeplex® Myc-TB]).

### Sample Processing and Laboratory Testing

***Smear Microscopy:*** Smear microscopy results were classified as negative, scanty (+), moderate (++), or high (+++) according to the standard WHO grading criteria^18^.

### Xpert Ultra

The Xpert Ultra assay (Cepheid, USA)^19^ was performed directly on 1.5 mL of clinical specimens in accordance with the manufacturer's protocol. Samples were treated with a 2:1 ratio of sample reagent to sputum, incubated, and subsequently loaded into cartridges for automated real-time PCR detection of *M. tuberculosis* and RIF resistance. The cycle threshold (Ct) value was recorded and categorized as <16 (indicative of a high bacillary load) or ≥16 (paucibacillary), based on the distribution of the samples.

### Line Probe Assays (LPA-1 and LPA-2)

First-line (MTBDRplus) and second-line (MTBDRsl) LPAs (Hain Lifescience, Germany)^20^, were performed directly on clinical samples using the GenoLyse® kit (Hain Lifescience, Germany). Hybridization results were interpreted visually. In instances of invalid or ambiguous results, DNA was re-extracted, and the assay was repeated. For LPA-1, resistance to rifampicin and isoniazid was assessed by detecting mutations in the *rpoB*, *katG*, and *inhA* genes. Heteroresistance was inferred when both wild-type and mutant bands were observed at the same locus. LPA-2 targeted mutations in *gyrA* and *gyrB* (associated with fluoroquinolones) as well as in *rrs* and *eis* (linked to injectable drugs).

### Phenotypic Drug Susceptibility Testing (MGIT)

Phenotypic DST was conducted using the BACTEC™ MGIT™ system (Becton Dickinson, USA)^21^, following the manufacturer's guidelines. The critical drug concentrations were as follows: streptomycin (SM) 1.0 μg/mL, isoniazid (INH) 0.1 μg/mL, rifampicin (RIF) 1.0 μg/mL, and ethambutol (EMB) 5.0 μg/mL for first-line drugs; and the WHO-recommended concentrations for second-line drugs^3^.

### Targeted Next-Generation Sequencing (T-NGS)

All samples underwent testing with the Deeplex® Myc-TB assay (Genoscreen, Lille, France)^22^. Libraries were prepared following Illumina protocols and sequenced on the Illumina MiSeq platform. This assay identifies resistance-associated mutations in genes related to first- and second-line drug resistance, assigns *M. tuberculosis* lineage and sublineage, and detects heteroresistant variants (defined as the coexistence of wild-type and mutant alleles at the same locus with allele frequencies between 5% and 94.9%, based on the WHO catalogue of mutations^23^). Mixed infections were inferred when lineage-specific single-nucleotide polymorphisms (SNPs) from two or more lineages were identified within a single sample.

### Definitions and Data Variables

Extracted variables included sample identifier (ID), year, and origin; smear microscopy grade; Ct values from Xpert Ultra; resistance profiles and mutation data from LPA-1, LPA-2, and MGIT; and T-NGS outputs encompassing mutation-level resistance, lineage classification, mixed infection status, and heteroresistance (assessed only for isoniazid-associated genes, as allele frequency data were exclusively available for *katG* and *inhA*).

### Statistical Analysis

Diagnostic performance metrics, including sensitivity, specificity, positive predictive value (PPV), negative predictive value (NPV), and accuracy, were calculated for Xpert Ultra and LPA assays, using MGIT as the reference standard. Associations between LPA accuracy and bacillary load were evaluated using smear microscopy and Ct categories. Categorical comparisons were conducted using Fisher’s exact test. The concordance between MGIT and T-NGS was assessed using Cohen’s kappa (κ) with 95% confidence intervals (CIs). Discordant and heteroresistant results were analyzed on a case-by-case basis. All analyses were performed using Stata 17 (StataCorp, USA).

### Ethical Considerations

This study received approval from the Research Ethics Committee of the Clementino Fraga Filho University Hospital (UFRJ) under approval number 3.893.965.

## RESULTS

### Characteristics of the Analyzed Samples

A total of 40 respiratory clinical samples were analyzed. Smear microscopy was positive in 37 samples (92.5%), of which 20 samples (50.0%) exhibited a high bacterial load (+++), 13 (32.5%) were categorized as (+), and 4 (10.0%) were categorized as (++). Only three samples (7.5%) were smear-negative.

T-NGS lineage assignment revealed a predominance of Latin American-Mediterranean strains. The most frequent sublineages included 4.1.2.1 (n=8; 20.0%), 4.3.4.1 (n=7; 17.5%), and 4.3.3 (n=5; 12.5%). Less common sublineages included 4.7, 4.3.4.2, and 4.2 (each n=3; 7.5%), as well as 4.3.2, 4.8, 4.1.1.3, and 4.3.4 (each n=1; 2.5%). Mixed infections were identified in seven samples (17.5%), including one sample with a minor lineage 2 (Beijing) population. The most common combinations involved lineage 4.1.2.1 with either lineage 4.3.3 or 4.3.4.1.

### Performance of Molecular Assays (Xpert Ultra and LPA-1)

Using MGIT as the reference, Xpert Ultra detected RIF resistance with a sensitivity of 91.7% (95% CI: 73.0-99.0%) and a specificity of 100% (95% CI: 73.5-100%). The PPV was 100%, the NPV was 85.7%, and the overall accuracy reached 94.4% (Table 1).

**TABLE 1:** Diagnostic performance of Xpert Ultra and LPA-1 for rifampicin and INH resistance detection, using MGIT as phenotypic reference (n=40).

Drugs	Sensitivity (95% CI)	Specificity (95% CI)	PPV (95% CI)	NPV (95% CI)	Accuracy (%) (95% CI)
Xpert Ultra (RIF)	91.7% (73.0-99.0)	100% (73.5-100)	100% (84.6-100)	85.7% (57.2-98.2)	94.4% (81.1-98.5)
LPA-1 (RIF)	96.2% (80.4-99.9)	78.6% (49.2-95.3)	89.3% (71.8-97.7)	91.7% (61.5-99.8)	90.0% (76.9-96.0)
LPA-1 (INH)	81.5% (61.9-93.7)	92.3% (64.0-99.8)	95.7% (78.1-99.9)	70.6% (44.0-89.7)	85.0% (70.9-92.9)

**RIF:** rifampicin; **INH:** isoniazid; **CI:** confidence interval; **PPV:** positive predictive value; **NPV:** negative predictive value.

LPA-1 demonstrated slightly higher sensitivity but lower specificity for rifampicin detection, with a sensitivity of 96.2% (95% CI: 80.4-99.9%) and a specificity of 78.6% (95% CI: 49.2-95.3%). The PPV and NPV were 96.2% and 78.6%, respectively, resulting in an overall accuracy of 90.0%. For isoniazid, LPA-1 exhibited high specificity (92.3%; 95% CI: 64.0-99.8%) but lower sensitivity (81.5%; 95% CI: 61.9-93.7%), yielding an overall accuracy of 85.0%.

### Effect of Bacillary Load on LPA-1 Performance

The accuracy of LPA-1 for rifampicin and isoniazid ranged from 90.0% to 100.0% in (+++) smears and decreased in paucibacillary specimens (Table 2). Among the 22 samples with available Ultra Ct values, low Ct values (<16) were associated with (+++) smear results, whereas Ct values ≥16 were observed only in smear-negative or low-grade positive samples (p = 0.001).

**TABLE 2:** Diagnostic performance of LPA-1 according to the grade of smear microscopy, using MGIT as phenotypic reference (n=40).

	Rifampicin			Isoniazid		
Smear Microscopy	Concordant	Discordant	Accuracy (95% CI)	Concordant	Discordant	Accuracy (95% CI)
(+++)	18	2	90.0% (69.9-97.2)	18	2	90.0% (69.9-97.2)
(++)	4	0	100% (51.0-100)	4	0	100% (51.0-100)
(+)	11	2	84.6% (57.8-95.7)	9	4	69.2% (42.4-87.3)
Negative	3	0	100% (43.9-100.0)	3	0	100% (43.9-100)

**CI:** confidence interval.

### Concordance Analysis (MGIT and T-NGS)

For rifampicin and isoniazid, MGIT showed substantial and moderate concordance with T-NGS (κ=0.647 and κ=0.561; p<0.001), with overall agreement of 85.0% and 83.8%, respectively (Table 3). For ethambutol, concordance was lower (κ = 0.258; observed agreement 76.9%). No phenotypically resistant isolates to amikacin or fluoroquinolones were detected by MGIT; the observed agreement with T-NGS was 81.25% in both comparisons, but κ was uninformative (~0) due to a lack of phenotypic variability.

**TABLE 3:** Agreement between MGIT and T-NGS for resistance detection to first- and second-line anti-tuberculosis drugs (n=40).

Drug	Observed Agreement	Expected Agreement	Kappa	Standard Error	Z	p-value
Rifampicin	85.0%	57.5%	0.647	0.153	4.21	< 0.001
Isoniazid	83.8%	63.0%	0.561	0.162	3.45	< 0.001
Ethambutol	76.9%	68.9%	0.257	0.159	1.62	0.053

**Z:** Z-score test for the null hypothesis of no agreement beyond chance.

### Concordance in Detecting Isoniazid Heteroresistance (LPA and T-NGS)

Isoniazid heteroresistance was detected by LPA in 14 samples (35.0%) but confirmed by T-NGS in three cases (7.5%), all involving *katG* (variant frequencies 19.6% - 94.9%). The remaining 11 samples exhibited variant frequencies ≥95%, indicating fixed resistance in sequencing, despite intermediate patterns observed in the LPA strips. One sample displayed the *inhA* promoter c-15t at 5.23%, near the detection limit (Table 4).

**TABLE 4:** Comparison of isoniazid heteroresistance classification by LPA and T-NGS (*katG* and *inhA* genes).

Sample ID	LPA Heteroresistance	T-NGS Gene	Mutation	VAF	T-NGS Classification
1510-18	Yes	KatG	S315T	99.27%	Fixed resistance
1750-18	Yes	KatG	S315T	99.37%	Fixed resistance
1670-18	Yes	KatG	S315T	99.61%	Fixed resistance
1940-18	Yes	KatG	S315T	98.83%	Fixed resistance
2439-18	Yes	KatG	S315T	100.00%	Fixed resistance
1970-18	Yes	KatG	S315T	94.56%	Heteroresistance
1668	Yes	KatG	S315T	99.29%	Fixed resistance
272-21	Yes	KatG	S315T	100.00%	Fixed resistance
475-21	Yes	KatG	S315T	98.75%	Fixed resistance
1238-21	Yes	KatG	S315T	96.55%	Fixed resistance
TRU21	Yes	KatG	S315T	100.00%	Fixed resistance
LPA73	Yes	KatG	S315T	19.60%	Heteroresistance
LPA201	Yes	KatG	S315T	91.24%	Heteroresistance
2442-18	Yes (*inhA*)	inhA/prom.	c-15t	5.23%	Heteroresistance

**LPA:** line probe assay; **VAF:** variant allele frequency; **T-NGS:** targeted next-generation sequencing.

### Mutations Associated with Discordant Results

Among the discordant drug susceptibility testing (DST) results, all cases of *ethambutol* (n = 5; 100%) were resistant according to T-NGS and susceptible according to MGIT, carrying *embB* mutations either strongly associated with resistance (M306V, M306I, G406A) or of uncertain significance (A451T). Regarding *isoniazid* (n = 8), T-NGS detected resistance in 62.5% of MGIT-susceptible samples, primarily involving *katG* S315T or mutations of uncertain significance (*katG* F129S, *ahpC* P44R); two samples were T-NGS-sensitive/MGIT-resistant, and one was LPA-sensitive/MGIT-resistant. For *rifampicin* (n = 6), 83.3% were T-NGS resistant/MGIT-susceptible, with *rpoB* mutations associated with resistance (H445N, S450W) or other mutations of uncertain significance or uncharacterized (P454S, A405T, K258E/S458L). Conversely, 16.7% of samples were MGIT-resistant/T-NGS-sensitive, exhibiting wild-type *rpoB* sequences (Table 5).

**TABLE 5:** Mutations associated with discordant results between assays.

Drug	Sample ID	Xpert Ultra	LPA	MGIT	T-NGS	Mutation (Gene: change)	Mutation Classification
Rifampicin	1126-21	Resistant	Resistant	Susceptible	Resistant	rpoB: H445N, S450W	Associated with resistance
Rifampicin	2442-18	Susceptible	Susceptible	Resistant	Susceptible	rpoB: wild-type	Wild-type
Rifampicin	LPA137	Resistant	Resistant	Susceptible	Resistant	rpoB: S450W	Associated with resistance
Rifampicin	LPA163	Susceptible	Susceptible	Susceptible	Resistant	rpoB: P454S	Uncertain significance
Rifampicin	LPA166	Susceptible	Susceptible	Susceptible	Resistant	rpoB: A405T	Uncharacterized
Rifampicin	LPA74	Susceptible	Resistant	Susceptible	Resistant	rpoB: K258E, S458L	Uncharacterized
Isoniazid	1126-21	NA	Susceptible	Susceptible	Resistant	katG: S315T	Associated with resistance
Isoniazid	1160-21	NA	Susceptible	Resistant	Susceptible	katG: wt / inhA: wild-type	Wild-type
Isoniazid	1511-18	NA	Susceptible	Resistant	Susceptible	katG: wt / inhA: wild-type	Wild-type
Isoniazid	172-20	NA	Susceptible	Resistant	Resistant	katG: F129S	Uncertain significance
Isoniazid	2652-HP	NA	Susceptible	Susceptible	Resistant	ahpC: P44R	Uncertain significance

## DISCUSSION

This study evaluated the performance of Xpert Ultra, LPA-1, and LPA-2 in detecting resistance to first- and second-line anti-tuberculosis drugs, utilizing MGIT as a reference method. Additionally, T-NGS was employed to identify resistance-conferring mutations and to clarify discrepancies in results.

Substantial agreement was found between MGIT and T-NGS for rifampicin (κ=0.65) and moderate agreement for isoniazid (κ=0.56), consistent with previous reports and reinforcing T-NGS as a reliable method for detecting mutations that are not consistently identified by phenotypic assays^23^ ^-^ ^24^. In contrast, agreement for ethambutol was only fair (κ=0.26), reflecting known challenges in phenotypic detection due to complex resistance mechanisms or variable critical concentrations^25^; most discordances involved embB mutations (M306V, M306I, G406A, A451T) detected by T-NGS but not by MGIT.

No phenotypically resistant isolates to fluoroquinolones or amikacin were identified by MGIT. While specificity was maintained, sensitivity could not be assessed in the absence of resistant phenotypes. Nevertheless, T-NGS revealed resistance-associated variants in *gyrA* and *rrs/eis*, highlighting the added value of sequencing for detecting second-line drug resistance.

Xpert Ultra demonstrated high sensitivity (91.7%) and specificity (100%) for rifampicin resistance compared to MGIT, reaffirming its role as a front-line diagnostic tool. Importantly, T-NGS clarified several "false-positive resistant" results from Xpert Ultra, which corresponded to disputed mutations in rpoB (e.g., H445N, S450W), aligning with prior findings^26^ and underscoring the importance of sequencing in clarifying cases of borderline resistance.

LPA-1 exhibited high sensitivity for rifampicin; however, it displayed lower specificity. Discrepancies often involved *rpoB* mutations, which may be variably detected by MGIT or correspond to silent or disputed mutations that are not fully covered by LPA probes^27^. For isoniazid, the lower sensitivity likely reflects undetected mutations outside the *katG* or *inhA* regions targeted by the assay^28^ ^-^ ^31^.

As anticipated, the performance of molecular assays was affected by bacillary burden: accuracy was highest in smear-positive (+++) samples and declined in paucibacillary specimens^30^; Ct data supported this trend, with Ct values <16 in most (+++) cases compared to Ct values ≥16 in smear-negative or low-grade samples^32^. Under field conditions, both LPA-2 and MGIT underestimated second-line resistance relative to T-NGS, consistent with reports of limited sensitivity for LPA-2^33^ ^-^ ^35^.

Mixed infections (17.5% in our cohort) and heteroresistance present significant diagnostic and clinical challenges. Mixed infections can obscure minority resistant subpopulations and yield false-susceptible results in both molecular and phenotypic assays^36^ ^-^ ^40^. Regarding heteroresistance, several LPA-classified heteroresistant cases were not confirmed by T-NGS, which may reflect the limitations of sequencing for low-frequency variants, hybridization artifacts, or methodological discrepancies between assays. This highlights the necessity of interpreting borderline resistance patterns within an integrated diagnostic algorithm.

Beyond analytical clarification, these discordances have treatment and programmatic implications. Undetected resistance can result in delayed effective therapy and continued transmission. Sequencing methods that detect minority variants enable earlier adjustments to treatment regimens and closer follow-u^p^ ^41^ ^-^ ^43^. Resistance identified solely by T-NGS in *embB*, *gyrA*, and *rrs* suggests an underestimation of resistance to ethambutol, fluoroquinolones, and amikacin by routine testing, which may delay the initiation of WHO-recommended regimens (such as bedaquiline or linezolid) or lead to suboptimal treatment combinations^12^ ^,^ ^41^. Thus, integrating T-NGS into reference laboratory workflows, particularly for complex or discordant cases, can enhance regimen selection, reduce time to effective therapy, and strengthen resistance surveillance in high-burden settings.

The lineage distribution observed (39/40 lineage 4; one lineage 2/Beijing) was consistent with expectations for South America^44^, and resistance mutations were predominantly located in *rpoB* and *katG*, with fewer in *inhA*, as reported elsewhere^45^ ^,^ ^46^. Overall, T-NGS proved instrumental in addressing disputed mutations, inferring resistance, and identifying heteroresistance and mixed infections, supporting WHO recommendations to include sequencing in diagnostic algorithms in high-burden/reference settings^12^ ^,^ ^41^.

Limitations of this study include the small sample size for evaluating second-line resistance and the absence of minimum inhibitory concentration (MIC) testing, which could aid in correlating mutation profiles with phenotypic resistance levels. Nevertheless, the inclusion of T-NGS provided comprehensive resistance data, highlighting key diagnostic gaps in current algorithms. Furthermore, because PPVs and NPVs depend on the underlying prevalence of DR-TB, the reported values are applicable to populations with a prevalence similar to that observed in this study and should be interpreted accordingly.

In summary, while Xpert Ultra and LPA-1 demonstrated high sensitivity for rifampicin detection, both tests exhibited limited specificity in the presence of disputed mutations. LPA-1 also showed reduced sensitivity for isoniazid due to limited mutation coverage. The performance of molecular tests decreased in paucibacillary samples, and mixed infections were common. T-NGS provided critical insights into undetected resistance, heteroresistance, and discordant cases, which should be strategically incorporated into DR-TB diagnostic workflows.

## Data Availability

Data are available from the corresponding author upon reasonable request and subject to ethical approval.
